# Universal Two-Component Dynamics in Supercritical Fluids

**DOI:** 10.1021/acs.jpcb.1c07900

**Published:** 2021-12-02

**Authors:** Peihao Sun, J. B. Hastings, Daisuke Ishikawa, Alfred Q. R. Baron, Giulio Monaco

**Affiliations:** † SLAC National Accelerator Laboratory, 2575 Sand Hill Road, Menlo Park, California 94025, United States; ‡ Materials Dynamics Laboratory, RIKEN SPring-8 Center, 1-1-1 Kouto, Sayo, Hyogo 679-5148, Japan; § Dipartimento di Fisica e Astronomia, Università di Padova, 35131 Padova, Italy; ∥ Physics Department, Stanford University, 382 Via Pueblo Mall, Stanford, California 94305, United States

## Abstract

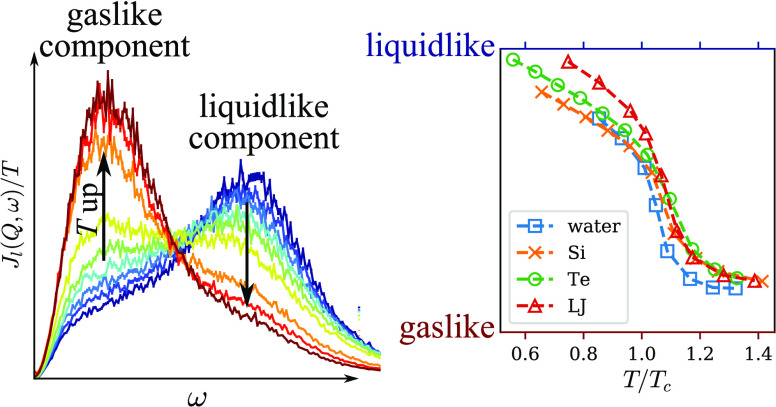

Despite the technological importance of supercritical fluids, controversy remains about the details of their microscopic dynamics. In this work, we study four supercritical fluid systems—water, Si, Te, and Lennard-Jones fluid—via classical molecular dynamics simulations. A universal two-component behavior is observed in the intermolecular dynamics of these systems, and the changing ratio between the two components leads to a crossover from liquidlike to gaslike dynamics, most rapidly around the Widom line. We find evidence to connect the liquidlike component dominating at lower temperatures with intermolecular bonding and the component prominent at higher temperatures with free-particle, gaslike dynamics. The ratio between the components can be used to describe important properties of the fluid, such as its self-diffusion coefficient, in the transition region. Our results provide an insight into the fundamental mechanism controlling the dynamics of supercritical fluids and highlight the role of spatiotemporally inhomogeneous dynamics even in thermodynamic states where no large-scale fluctuations exist in the fluid.

## Introduction

In the past few decades, supercritical fluids have attracted renewed interest due to their applications in a wide range of chemical and materials processing industries.^1^ Most interesting applications of supercritical fluids fall in the region close to the critical point.^1,2^ There, the fluids exhibit unique properties combining the advantages of liquids (e.g., high densities) and gases (e.g., high diffusivities), and these properties are highly tunable with relatively small changes in temperature, *T*, and pressure, *P*.^2^ Thus, it is important to understand these properties and their dependence on the thermodynamic state.

Thanks to many years of research, the thermodynamics of supercritical fluids, which is based on their macroscopic properties, has become well understood. In particular, the concept of the Widom line has been introduced to refer to the line of maxima of a given response function, such as the isobaric heat capacity, *C*_P_.^3^ Although not a rigorous separatrix between liquid and gas states,^4^ the Widom line indicates rapid changes in the thermodynamic properties of supercritical fluids, especially in the near-critical region. Around the Widom line, a crossover between liquidlike and gaslike properties is expected for the fluid.^5^

The picture is less clear when it comes to molecular-scale dynamics of supercritical fluids, which should reveal the microscopic mechanism behind many of the macroscopic properties. One of the first systematic studies on this topic was done by Simeoni et al.^6^ Using classical molecular dynamics (MD) simulations supported by inelastic X-ray scattering (IXS) data, they observed a crossover in the deep supercritical region along an extension of the Widom line.

Our previous work^7^ focused instead in a region close to the critical point, where the Widom line is very clear. We used both IXS measurements and MD simulations to study the intermolecular dynamics of supercritical water in the region 0.9 < *P*/*P*_c_ < 2.3, 0.6 < *T*/*T*_c_ < 1.2, where *P*_c_ and *T*_c_ are the critical pressure and temperature. Contrary to previous approaches,^6,8^ we found that the intermolecular dynamics at a given *P*,*T* state cannot be consistently described using models developed for liquids, but instead can be decomposed into two components—a high-frequency component associated with the stretching mode between hydrogen-bonded molecules and a low-frequency component representing free-particle motions. With changing thermodynamic states, it is the ratio between the two components that changes, with a rapid crossover observed near the Widom line. However, remnants of both components can be found on either side of the Widom line.

It is natural to ask whether the observed two-component dynamics is specific to water, whose liquidlike dynamics arises from hydrogen bonds, or can be generalized to other supercritical fluids. In this work, we aim at answering this question by studying the potentials representing four different supercritical fluid systems—water, Si, Te, and Lennard-Jones (LJ) fluid—via classical MD simulations. Even though these systems have very different interatomic potentials (see the Methods section), the two-component behavior is universal in their molecular dynamics. Moreover, we find evidence to associate the liquidlike component with the degree of intermolecular bonding and the gaslike component with dynamics similar to that in an unbonded, free gas state. As in the case of water, a fast change in the ratio between the two components marks the dynamical crossover, but both components exist on either side of the transition. The fraction of the components can also be used to describe the transport properties of the fluid, such as its self-diffusion coefficient.

## Methods

### Simulation Details

In this study, we investigate four fluid systems with different potential models:

(1) Water, with the TIP4P/2005 potential.^9^ This potential includes a Lennard-Jones (LJ) interaction between oxygen sites and long-range Coulomb force between all charged sites.
(2) Si, with the Stillinger–Weber (SW) potential.^10^ This potential includes pairwise interactions as well as three-body interactions, both short-ranged (cutoff at 3.771 Å). The three-body interaction term favors local tetrahedral ordering.
(3) Te, with an analytical bond-order potential (BOP).^11^ This potential considers the effect of bond orders, which are functions of the local environments of the atoms, on the bond energy.
(4) LJ fluid, with the shifted-force (sf) potential

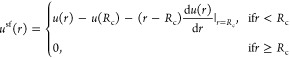
1where *r* is the distance between interacting atoms, *R*_c_ is the cutoff distance, and
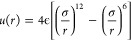
2is the standard 12-6 potential. ϵ and σ are energy and distance units, respectively. Other units for the LJ fluid can be expressed in terms of ϵ, σ, and the atomic mass *M*. For example, the unit for time is . In this work, we set *R*_c_ = 2.5σ.

The MD simulations are carried out using the LAMMPS simulation package.^12^ The simulation box contains 2880 molecules for water and 4000 atoms for Si, Te, and LJ fluid. We use NPT ensembles, with a Nosé–Hoover thermostat and barostat. The damping constants are 1 ps for water, Si, and Te, and 1 τ for LJ. After equilibration at each *P*,*T* state, the simulation is run for 1 ns (with 1 fs time steps) for water, 0.4 ns (with 1 fs time steps) for Si, 1 ns (with 2.5 fs time steps) for Te, and 1000 τ (with 0.001 τ time steps) for LJ.

### Critical Parameters

**Figure 1:**
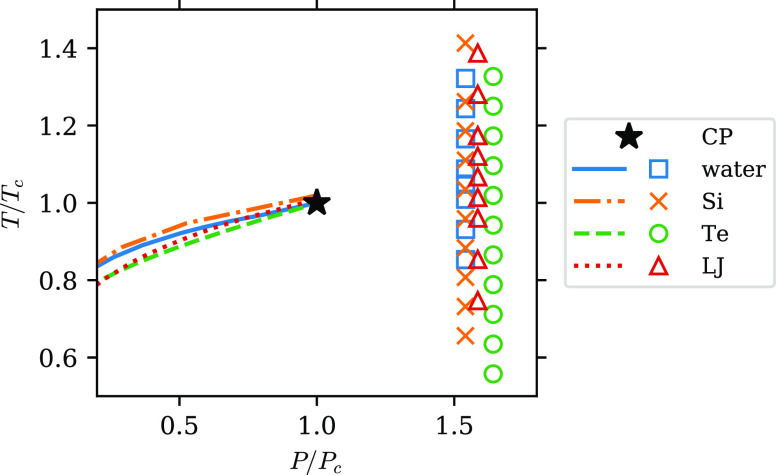
*P*–*T* phase diagram of the systems in reduced units. The lines show the liquid–vapor coexistence line for the systems (see refs (16−18) for water, Si, and LJ, and the Supporting Information for Te), which terminate at the critical point (CP, black star). The symbols show the thermodynamic states included in this study.

Table 1 presents the critical point parameters for the fluid systems in this study. The TIP4P/2005 model for water, the SW model for Si, and the LJ fluid model are well studied, and their critical parameters can be found in the literature. The critical point parameter for the BOP Te model is determined using a direct MD simulation method;^13^ more details are provided in the Supporting Information. Most of the results below focus on the temperature dependence of the properties of the fluid along an isobar *P* ≈ 1.6*P*_c_; the exact value of *P* for each system is listed in the last column in Table 1. Figure 1 shows the (reduced) *P*–*T* phase diagram of all of the systems, as well as the thermodynamic states simulated in this study. We note that, as mentioned in the Discussion and Conclusions section, the two-component phenomenon is not an anomaly arising from large-scale critical fluctuations, and the isobars taken are sufficiently away from the critical point. Therefore, the results in this study are robust against errors in the critical point parameters.

**Table 1:** Critical Point Parameters for the Models Used in This Study and the Isobar Pressure *P*^a^

model	*T*_c_	*P*_c_	ρ_c_	*P*	*T* range
water^14^	640 ± 16	146 ± 7	0.337 ± 0.008	225	546–846
Si^15^	7925 ± 250	1850 ± 400	0.75 ± 0.10	2850	5200–11 200
Te	2080 ± 40	530 ± 40	2.17 ± 0.04	870	1160–2760
LJ^16^	0.937ϵ/*k*_B_	0.0820ϵ/σ^3^	0.320σ^–3^	0.13ϵ/σ^3^	0.7–1.3ϵ/*k*_*B*_

a The units for water, Si, and Te are: *T*_c_ in K, *P*_c_ and *P* in bar, and ρ_c_ in g/cm^3^. The last column shows the temperature range investigated for each system.

## Results

### Two-Component Dynamics

The molecular dynamics of fluids is usually described by the dynamic structure factor, *S*(*Q*, ω), which measures the correlation of density fluctuations in wavenumber (*Q*) and frequency (ω) space.^19^ It is defined as
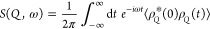
3where angular brackets indicate the ensemble average and ρ_*Q*_(*t*) = ∑_*n* = 1_^*N*^*e*^*i***Q**·**r**_*n*_(*t*)^/√*N* is the density in *Q*-space at time *t*, with **r**_*n*_(*t*) being the position of the *n*th atom. In this paper, we take the classical limit. *S*(*Q*, ω) is one of the most important functions to describe the molecular dynamics of fluids, as it contains all of the relevant information on the dynamics of the system.^19^ Moreover, at wavenumbers approaching intermolecular scales (*Q* ∼ Å^–1^), *S*(*Q*, ω) can be directly measured using inelastic neutron and X-ray scattering.^19,20^

The dynamics in different thermodynamic states can be conveniently compared using the longitudinal current correlation *J*(*Q*, ω), defined by replacing the density ρ_*Q*_(*t*) in eq 3 with the longitudinal current *j*_*Q*,*l*_(*t*) = ∑_*n* = 1_^*N*^*v*_*n,l*_(*t*)*e*^*i***Q**·**r**_*n*_(*t*)^/√*N*. Here, *v*_*n*,*l*_(*t*) denotes the velocity of the *n*th atom along the direction of **Q**. It bears a simple relation to *S*(*Q*, ω)^19^
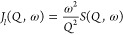
4*J*(*Q*, ω) obeys the classical sum rule^19^
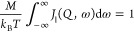
5where the pre-factor contains only the molecular mass *M*, the Boltzmann constant *k*_B_, and the temperature *T*, all of which are known constants for the simulation. This provides a simple way to normalize and compare the spectra for different thermodynamic states.

**Figure 2:**
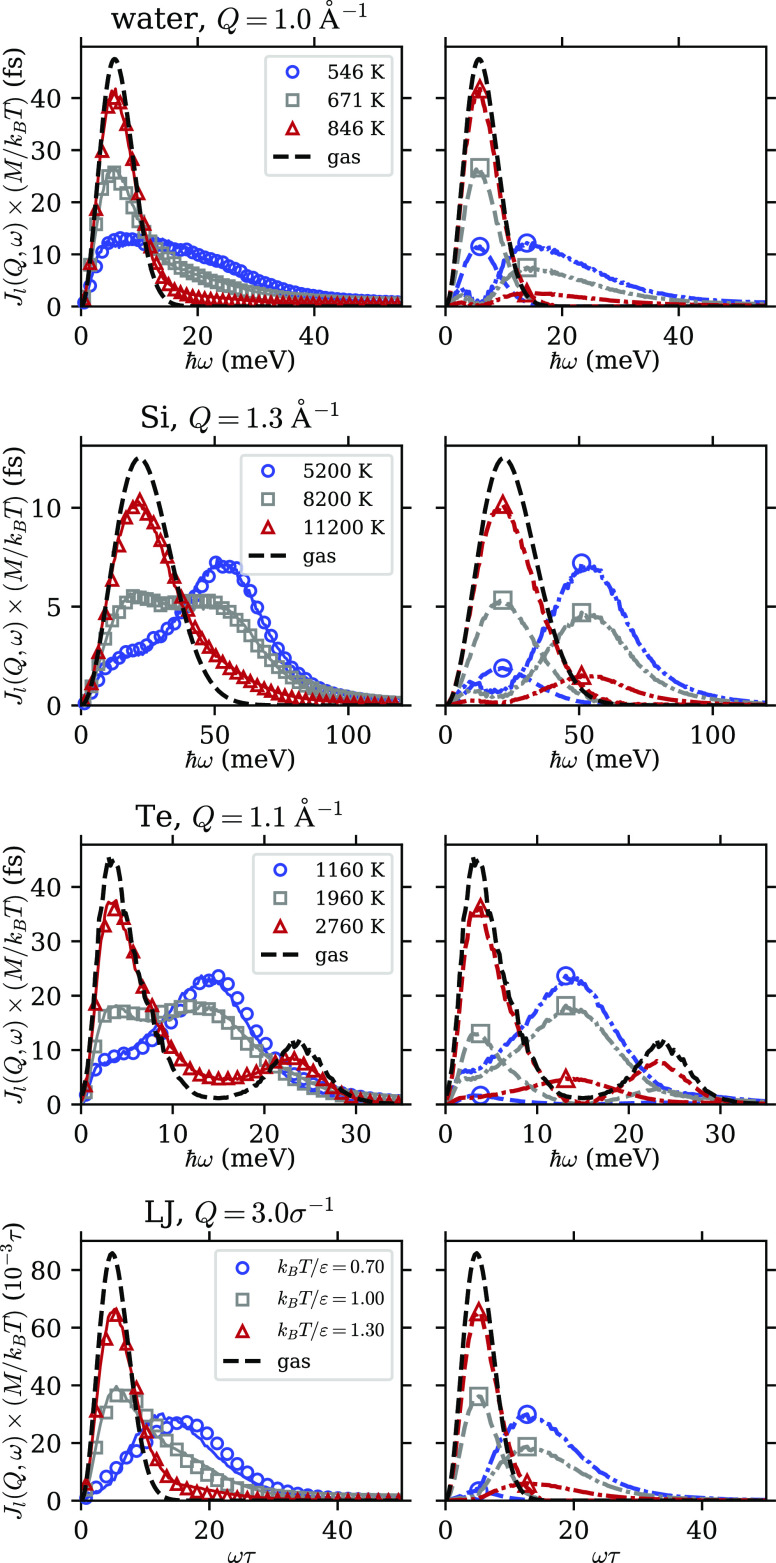
Two-component behavior in the longitudinal current correlation function. Each row shows a different fluid system. The left columns show data from simulation (symbols) along with the NMF fit (solid lines). For each system, we choose three states along an isobar *P* ≈ 1.6*P*_c_: a low-temperature, liquidlike state (blue circles), an intermediate state in the crossover region (gray squares), and a high-temperature, gaslike state (red triangles). The right column shows the L component (dash-dotted lines) and G component (dashed lines) obtained from NMF, and the peak positions are marked by corresponding symbols. For reference, we show in both columns the gas limit as black dashed lines without symbols. σ and ϵ are LJ units.

With the help of this normalization, the two-component behavior in the fluid systems becomes clear. This can be seen in Figure 2, where the symbols on the left column show the normalized spectra, *J*_l_(*Q*, ω) ×(*M*/*k*_B_*T*), obtained from MD simulations. Each row presents one of the four fluid systems in this study—water, Si, Te, and LJ fluid—as indicated. For each system, three temperature points are taken along an isobar of *P* ≈ 1.6*P*_c_ as indicated in Table 1: a low-temperature state (blue circles), an intermediate-temperature state (gray squares), and a high-temperature state (red triangles). The *Q* value is chosen to be ∼0.5*Q*_m_, where *Q*_m_ is the position of the first peak in the structure factor *S*(*Q*); in real space, this *Q* corresponds to approximately twice the average intermolecular distance. We note that the same two-component phenomenon can be observed at other *Q* values at least in the range of 0.3*Q*_m_ to 0.8*Q*_m_, as was also the case in our previous work.^7^

The black lines in Figure 2 show the spectra expected of the gas state. For water, Si, and LJ fluid, this is taken to be the free-particle limit, assuming simply a Maxwell–Boltzmann velocity distribution with no interaction^7,19^
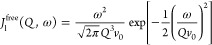
6where  is the thermal velocity. The temperature is taken to be the same as the high-*T* state, although small changes in *T* lead only to a slight shift (∝√*T*) in the peak position and do not appear to significantly influence the results below. For Te, the gas phase is diatomic (i.e., it consists of Te_2_ dimers), so there is an additional peak around 23 meV corresponding to the dimer stretching mode (see the Supporting Information for more details). Hence, a simple expression cannot be obtained for *J*_l_^free^(*Q*, ω), and we use instead a low-*P* spectrum at 100 bar, 2760 K, where the density is only 0.109 g/cm^3^ compared to the critical density of 2.17 g/cm^3^. It can be seen that the high-*T* spectrum is close to the gas state for all systems.

From these plots, it is clear, particularly for Si and Te, that the intermediate state contains features of both the low- and high-temperature spectra as in the case of water.^7^ Specifically, the intermediate spectrum in Si shows both the peak around 55 meV, which is prominent in the low-*T* state and the peak around 20 meV, which dominates the high-*T* state, and similarly for Te (including the dimer oscillation peak around 23 meV). In the case of the LJ fluid, even though we do not observe two distinct peaks, the intermediate-temperature spectrum can still be interpreted as a linear combination of the high- and low-temperature states. In addition, as will be shown below, this interpretation can be used to predict other properties of the LJ fluid in the same way as for the other systems. Therefore, our results show that there is a universal two-component behavior in the supercritical fluids under study.

### NMF Analysis and the Liquidlike-to-Gaslike Transition

To describe the spectra quantitatively, a method is needed to extract the two components. To our knowledge, however, no existing theory can adequately describe the two-component phenomenon and provide a model to fit the data. Therefore, we adopt the non-negative matrix factorization (NMF) method^21^ used in our previous study,^7^ which provides a model-free way to extract the components in the spectra. Mathematically, we optimize the decomposition
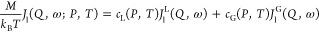
7where *J*_l_^L^(*Q*, ω) and *J*_l_^G^(*Q*, ω) are the L and G components dominating in the liquidlike (low *T*) and gaslike (high *T*) states, respectively; the shapes of these components are assumed to be independent of *P* and *T*. The pressure and temperature dependence of the normalized spectra are captured entirely in the coefficients *c*_L_(*P*, *T*) and *c*_G_(*P*, *T*). When fitting, we include all temperatures along the isobar and add the gas state as well. It also turns out that spectra from different *Q*’s can be fit together, resulting in the same coefficients *c*_L_(*P*, *T*) and *c*_G_(*P*, *T*). Because we are interested in molecular-scale dynamics, in this work, we typically use data from 0.3*Q*_m_ to 0.8*Q*_m_, which corresponds to length scales on the same order as the average intermolecular distance. Small changes in the *Q* range used for fitting do not have a significant influence on the results below. When *Q* < 0.3*Q*_m_, the data tend to be noisier because of the finite system size and energy resolution.

Results of the NMF decomposition are shown in Figure 2. On the left column, the solid lines show the NMF fit (sum of the components), which agrees well with data (symbols); on the right column, the G and L components are shown as dashed and dash-dotted lines, respectively, with the corresponding symbols indicating their respective peak positions. In all systems, the G component is close in shape to the gas-state spectrum and the L component peaks at a higher frequency. With increasing temperature, the spectral weight shifts from the L component to the G component, leading to a liquidlike-to-gaslike transition.

Because of the sum rule, eq 5, we normalize the L and G components as well so that ∫_–∞_^∞^*J*_l_^L,G^(*Q*, ω)dω = 1. As a result, *c*_L_ + *c*_G_ = 1, so we may interpret *c*_L_ and *c*_G_ as the fraction of the L and G components. If we now define the parameter *f* ≡ *c*_L_, it can be seen from eq 7 that the spectral evolution is captured entirely by the single parameter *f* as a function of *P* and *T*, and any dynamical crossover on an isobar should show up when plotting *f*(*T*).

**Figure 3:**
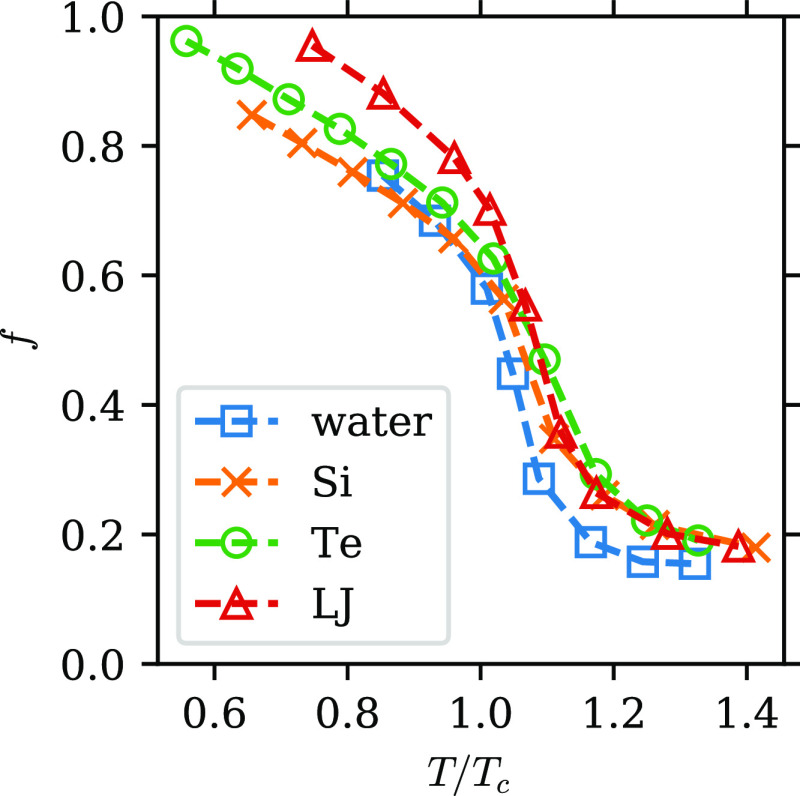
Dependence of the fraction of the L component, *f*, on reduced temperature *T*/*T*_c_ along the isobar *P* ≈ 1.6*P*_c_. Blue squares: water; yellow crosses: Si; green circles: Te; red triangles: LJ fluid. Dashed lines are a guide to the eye.

**Figure 4:**
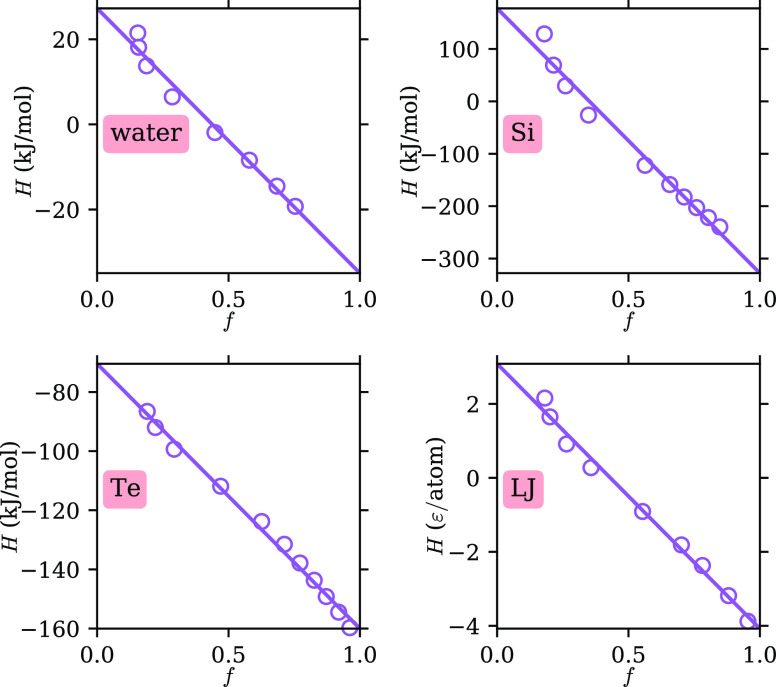
Relation between the enthalpy, *H*, and the parameter *f*. Data are shown as empty circles, and the linear fits are shown as solid lines. ϵ is the LJ energy unit (see Methods).

Therefore, in Figure 3, we present *f* as a function of reduced temperature *T*/*T*_c_ for all four systems. The overall shape and value of the curves are very similar for all of the systems. This is consistent with van der Waals law of corresponding states^22^ and provides evidence for the universality of the two-component behavior among supercritical fluids. In particular, all curves show an “S” shape with a rapid decrease slightly above *T*/*T*_c_ = 1. The position of the fast change in *f* agrees well with the expected location of the Widom line. To show this, we plot in Figure 4 the enthalpy, *H*, against the parameter *f*. The former can be easily obtained from MD simulations. An approximately linear relation can be seen between *f* and *H* for all systems, with linear fits shown as solid lines. Because the isobaric heat capacity, *C*_P_, is the derivative of *H* with respect to temperature along the isobar, the linearity between *f* and *H* implies that |d*f*/d*T*| peaks at roughly the same temperature as *C*_P_, i.e., near the Widom line. In other words, the dynamics change most rapidly around the Widom line. We note that although the Widom line here has a specific definition (*C*_P_ maximum along an isobar), in the near-critical region, it is expected to lie close to the Widom lines obtained by other definitions as well. For example, in the case of water, it has been shown that the rapid changes in *f* are close to the Widom lines with several different definitions.^7^

### L Component and Intermolecular Bonding

Having established above that the G component corresponds to the gas state, we now turn to the physical origin of the L component. In the case of water, our previous work^7^ has provided evidence that this component is related to the O–O stretching motion between hydrogen-bonded molecules. Therefore, it is reasonable to hypothesize that the L component in the other systems is related to intermolecular bonding as well.

To investigate this, it is necessary to define “bonding” for these systems. Because the LJ fluid has only a pairwise interaction that depends solely on the interatomic distance, it is natural to define a cutoff distance *R*_b_, below which a pair is considered bonded. In the following, we take *R*_b_ = 1.6σ, close to the first minimum in the radial distribution function *g*(*r*) in the low-temperature state at *T* = 0.7ϵ/*k*_B_, *P* = 0.13ϵ/σ^3^ (see the Supporting Information for details on *g*(*r*)).

The cases of Si and Te are in principle more complicated. Unlike water, whose hydrogen bonds can be defined by the geometry and/or the interaction energy between two molecules, Si and Te contain interaction terms that involve three or more atoms (see the Methods section). To our knowledge, there is no established way to define “bonding” in these systems. Hence, we simply define two atoms to be bonded if they are closer than a cutoff distance *R*_b_. As in the case of the LJ fluid, *R*_b_ is chosen to be around the first minimum in the radial distribution function for the low-temperature state, which is about 3.5 and 4.2 Å for Si and Te, respectively (see the Supporting Information).

**Figure 5:**
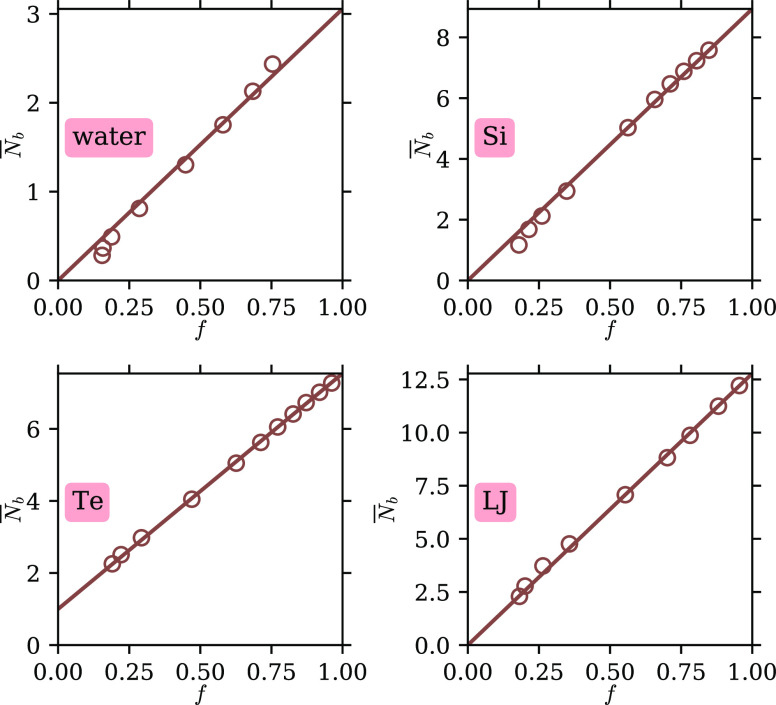
Relation between the number of bonds per atom (or water molecule), *N̅*_b_, and the parameter *f*. Data are shown as empty circles. For water, Si, and LJ, the data are consistent with an intercept of *N̅*_b_ = 0 at *f* = 0, as the solid lines show. For Te, the data are consistent with an intercept of *N̅*_b_ = 1 at *f* = 0 shown by the solid line.

In Figure 5, the circles show the average number of bonds each atom (or water molecule) has, *N̅*_b_, plotted against the parameter *f*. For water, as in our previous work, we use a common definition for hydrogen bonding: two molecules are hydrogen-bonded if their O–O distance is less than 3.5 Å and the O···O–H angle is less than 30°.^23,24^ For Si, Te, and LJ, we use the cutoff distance definition mentioned above. The data show very good linearity between *N̅*_b_ and *f*. Moreover, for water, Si, and LJ, the data are consistent with a zero intercept at *f* = 0, as the solid lines show. For Te, as mentioned above and shown in more detail in the Supporting Information, the gas state consists of Te_2_ dimers, so we expect each atom to have exactly one bond. Indeed, the data are consistent with an intercept of *N̅*_b_ = 1 at *f* = 0, as the solid line shows. These results strongly support that the L component, which dominates in low-temperature, liquidlike states, is directly related to intermolecular bonding for all systems studied. In the gas state, little to no bonding remains, and the L component disappears. We note that for water, using other hydrogen-bonding definitions with various levels of strictness does not alter the conclusion, and for Si, Te, and LJ, the conclusion is robust against changes in the cutoff distance being used up to at least 10% (see the Supporting Information).

### Application: Modeling the Self-Diffusion Coefficient

Our results above have provided evidence for the two-component dynamical behavior and have shown that *f* is a descriptor for the microscopic dynamics in the liquidlike to gaslike crossover. Since the microscopic dynamics is closely related to the macroscopic transport properties, there should be a close relation between *f* and transport properties as well. Below we show one such example.

One of the most important transport properties for supercritical fluids, especially for industrial applications, is the self-diffusion coefficient, *D*. This quantity can be easily obtained from MD simulations using the mean-square displacement^19^
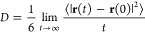
8where **r**(*t*) is the position of a given particle at time *t*. Here, angular brackets denote the ensemble average. For water, we use the position of the O atom. The simulation times are long enough to reach the *t* → ∞ limit. Alternatively, *D* can be obtained using the velocity autocorrelation function^19^
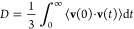
9where **v**(*t*) is the velocity of a given particle at time *t*. The results from the two methods agree within 5%.

Earlier work^5^ found that the self-diffusion coefficient for supercritical water appeared to follow an Arrhenius equation in the liquidlike and the gaslike region along each isobar. A dynamical crossover was found in between, but no specific model was given to describe it. Here, we propose a model in which the parameter *f* is used to describe this transition.

A good model should reduce to the observed dependence in the gaslike and liquidlike limits. In the limit of a dilute gas, it is well known^25,26^ that *D* has a power-law dependence on either the temperature *T* or the density ρ (*T* and ρ are inversely related on an isobar by the ideal gas law). Since in this limit the density should be proportional to the number of bonds per atom, which is in turn proportional to *f*, we expect *D* to have a power-law dependence on *f* as well. In the dense liquid limit, *D* is often described instead by the free volume model:^27,28^*D* ∝ exp(−*A*_v_/*V*_f_), where *A*_v_ is a constant and *V*_f_ is the free molecular volume (i.e., the average volume per molecule in excess of its van der Waals volume). Under the framework of our two-component dynamics description, we draw an analogy between the free volume, *V*_f_, and the fraction of the gaslike component, 1 – *f*. This can be justified by noting that, as shown above, the gaslike dynamic component corresponds to free-particle-like diffusive motions in the fluid. Therefore, in the dense liquid limit, we expect *D* ∝ *e*^–*A*/(1–*f*)^, where *A* is a constant.

**Figure 6:**
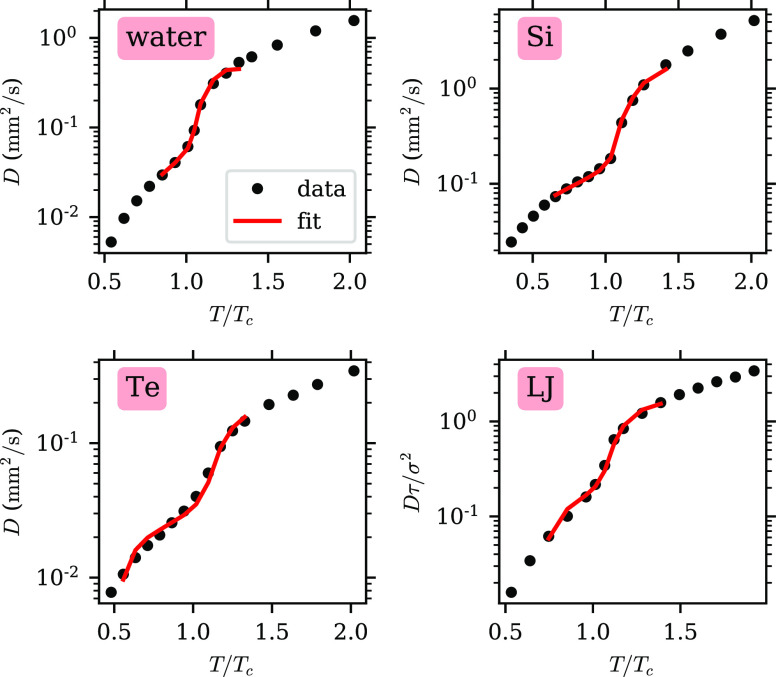
Self-diffusion coefficient, *D*, plotted against reduced temperature along an isobar *P* ≈ 1.6*P*_c_. The MD data are shown as black dots. The red lines show the fits using eq 10 over the range, where *f* is available through the two-component analysis.

Combining the two limits, we build the following model for the self-diffusion coefficient
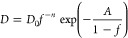
10where *D*_0_, *n*, and *A* are constants. In the gaslike limit, *f* → 0, so *D* → *D*_0_*f*^–*n*^*e*^–*A*^ ∝ *f*^–*n*^, i.e., it shows the expected power-law dependence. In the liquidlike limit, *f* → 1, so *D* → *D*_0_*e*^–A/(1–*f*)^ in line with the discussion above. We use eq 10 to fit the data with *D*_0_, *n*, and *A* as fit parameters, and the results are shown in Figure 6. The model is able to fit the data well, including the crossover region near the Widom line, where *D* increases rapidly with temperature. Values of the fit parameters are shown in Table 2. Except for Te, the exponent *n* in the gas limit is similar to literature values: *n* = 1.5 from the Chapman–Enskog theory,^26^ and *n* = 1.823 for nonpolar systems according to Slattery and Bird’s fit for experimental data.^25^ Note that, as mentioned above, on an isobar, we expect *f* ∝ ρ ∝ *T*^–1^, so we can rewrite expressions in the literature in terms of *f*. Through this example, we show that *f* can be used to describe macroscopic transport properties across the liquidlike-to-gaslike transition, connecting the limits of a dense liquid and a dilute gas. Given the proportionality between *f* and the number of bonds, *N̅*_b_, eq 10 may also be rewritten in terms of *N̅*_b_ and expanded to cover a wider range of thermodynamic states. This can be grounds for future investigations.

**Table 2:** Fit Parameters for the Self-Diffusion Coefficient Model, Eq 10^a^

system	ln *D*_0_	*n*	*A*
water	–3.16 ± 0.28	1.38 ± 0.12	0.181 ± 0.075
Si	–2.66 ± 0.11	1.84 ± 0.07	0.034 ± 0.021
Te	–3.83 ± 0.08	1.22 ± 0.08	0.033 ± 0.006
LJ	–1.93 ± 0.10	1.42 ± 0.08	0.044 ± 0.008

a *D*_0_ is in units of mm^2^/s (for water, Si, Te) or σ^2^/τ (for LJ).

## Discussion and Conclusions

To demonstrate the universality of the two-component phenomenon, we have chosen in our study four systems containing very different interatomic interactions (see the Methods section for more details)—the simple pairwise LJ potential, TIP4P/2005 water^9^ with long-range Coulomb forces, Stillinger–Weber (SW) silicon^10^ with a three-body term favoring local tetrahedral coordination, and tellurium bond-order potential,^11^ where the gas phase is diatomic. The appearance of the two-component dynamics in all systems shows that this phenomenon is not specific to the local bonding mechanism but common among several supercritical fluid systems. Consequently, any theory describing the molecular-scale dynamics of supercritical fluids, particularly the crossover between liquidlike and gaslike behavior, should take into account the existence of at least two components in the dynamics.

We note that the two-component phenomenon is not an anomaly arising from large-scale critical fluctuations since the thermodynamic states in this study are sufficiently far away from the critical point and no such large-scale fluctuations are observed in our simulations. Instead, our results suggest the presence of spatiotemporally heterogeneous dynamics on the molecular scale, reflecting unbounded and bounded particle motions. Notably, a recent work^29^ using machine learning on local structural information has also found the existence of molecular-scale heterogeneities in supercritical LJ fluids. Because of this, the two-component phenomenon is not expected to appear in the long-wavelength (low *Q*) limit. This has not been explored in our study by the low *Q* cutoff around 0.3*Q*_m_ as mentioned in the Results section. Nonetheless, macroscopic quantities are influenced by their microscopic mechanisms and, as shown above, the use of the two-component model for the molecular dynamics can help build a more fundamental understanding of macroscopic properties such as the diffusion coefficient.

We mention here another dynamical crossover proposed in the literature, the “Frenkel line”, which separates the supercritical region into “rigid” and “nonrigid” fluids depending on the relaxation time of the system.^30^ The underlying assumption there is that a single relaxation time describes the dynamics of all of the fluid. Here, we have shown, at least in the near-critical region we have investigated, that the dynamics is spatiotemporally heterogeneous. Thus, it is not appropriate to describe the dynamics as purely liquidlike or gaslike, but rather a combination of both. In our previous work on supercritical water^7^ including both experimental and simulation results, no significant change was observed near the proposed Frenkel line position. However, we have not investigated the deep supercritical region, where the Frenkel line might also exist;^30^ this may be a subject for future studies.

A limitation of our methodology using the NMF decomposition is the assumption that the shapes of the components do not change with the thermodynamic state. This of course does not work at all temperatures and pressures; for example, going to extremely high temperatures, the free-particle limit will be broadened according to eq 6. However, the fact that the NMF fit shown in Figure 2 works well indicates that this assumption is valid over the temperature range under study, around 0.6*T*_c_ to 1.4*T*_c_. As mentioned in the Introduction section, this range around the critical point is the most interesting for applications. A more rigorous theory taking into account the change in the shape of the components may be able to describe a wider range of thermodynamic conditions.

We emphasize that one interesting point of our approach is that it can be checked against scattering experiments, for example, high-resolution inelastic X-ray scattering.^20^ These experiments directly measure the dynamical structure factor, *S*(*Q*, ω),^19,20^ and the simple relation given by eq 4 connects it to *J*_l_(*Q*, ω). The *J*_l_(*Q*, ω) spectra are all that is needed for the two-component analysis and the extraction of the parameter *f*. Thus, *f* is a descriptor of microscopic dynamics that is experimentally accessible and, as shown above, it is connected with various other properties of the fluid. In our previous work,^7^ we have indeed used inelastic X-ray scattering to measure the molecular dynamics of supercritical water and found excellent agreement between experimental data and MD simulation results. Similar measurements can be done on other supercritical fluid systems as well to verify experimentally the universality of the two-component phenomenon found in this study. We note that, while the TIP4P/2005 water potential and the LJ potential have been shown to reproduce well experimental data on the dynamics of supercritical water^7^ and argon,^31^ the Si and Te potentials used in this study have not been optimized or checked against experimental data in the supercritical region since no data is yet available.

This universality and the close relation between intermolecular bonding and the L component is reminiscent of the well-known lattice gas model,^32,33^ which forms the basis connecting the liquid–gas critical point to the 3D Ising universality class. In the lattice gas model, a liquid-to-gas transition takes place with the breaking of bonds, which is similar to the behavior of *f* and its connection to intermolecular bonding found in our study. Furthermore, we note that both in the lattice gas model and in our two-component analysis, the liquidlike-to-gaslike transition happens gradually with a continuous loss of bonds. Therefore, our study suggests that the understanding of supercritical fluids based on the lattice gas model may be extended into the description of their molecular dynamics as well.

In conclusion, our results show that the two-component phenomenon in the molecular dynamics, previously observed in supercritical water,^7^ is universal among several supercritical fluid systems with different intermolecular interactions. While the gaslike (G) component corresponds to free-particle motion in a dilute gas, the liquidlike (L) component can be associated with intermolecular bonding (a generalization of hydrogen bonding in the case of water). These observations are shown to have important implications for transport properties such as the self-diffusion coefficient, particularly in bridging the liquidlike-to-gaslike transition, which is relevant to industrial applications.

## Supporting Information Available

Dimer gas phase of the Te BOP potential; simulation results to determine the critical parameters of the Te BOP potential; components retrieved by NMF fit and the dispersion relation; hydrogen-bond definitions; and the bonding definition for Si, Te, and LJ (PDF)
