# Supplementary material for: Universal Two-Component Dynamics in Supercritical Fluids
Source: J Phys Chem B. 2021 Dec 2;125(49):13494–501. doi: 10.1021/acs.jpcb.1c07900 (PMC8686117; doi:10.1021/acs.jpcb.1c07900)
Supplement: Supplementary file 1 — jp1c07900_si_001.pdf [file jp1c07900_si_001.pdf]

Universal Two-Component Dynamics in Supercritical Fluids (Supporting Information)

Peihao Sun,[†,§] J. B. Hastings,[†] Daisuke Ishikawa,[‡] Alfred Q. R. Baron,[‡] and Giulio Monaco[*,¶]

†*SLAC National Accelerator Laboratory, 2575 Sand Hill Rd, Menlo Park, CA 94025, USA*

‡*Materials Dynamics Laboratory, RIKEN SPring-8 Center, 1-1-1 Kouto, Sayo, Hyogo 679-5148, Japan*

¶*Dipartimento di Fisica e Astronomia, Università di Padova, 35131 Padova, Italy*

§*Stanford University Physics Department, 382 Via Pueblo Mall, Stanford, CA 94305, USA*

E-mail: giulio.monaco@unipd.it

Tellurium gas phase

In the main text we mentioned that the gas phase for the Te bond-order potential (BOP)[1] consists of dimer molecules. In Fig. S1 we show a snapshot of a dilute gas state produced using the VMD visualization program.[2] A bond is shown for each pair of atoms closer than 4.2 Å, same as defined in the main text. We can see that the vast majority of the atoms form dimers; very occasionally, a monomer or a cluster of more than two atoms can be seen. Although it is not the purpose of our work to accurately reproduce all experimental parameters of fluid tellurium, it should be noted that tellurium vapor is known to be diatomic (Te_2), with a disassociation energy around 61 kJ/mol.[3]

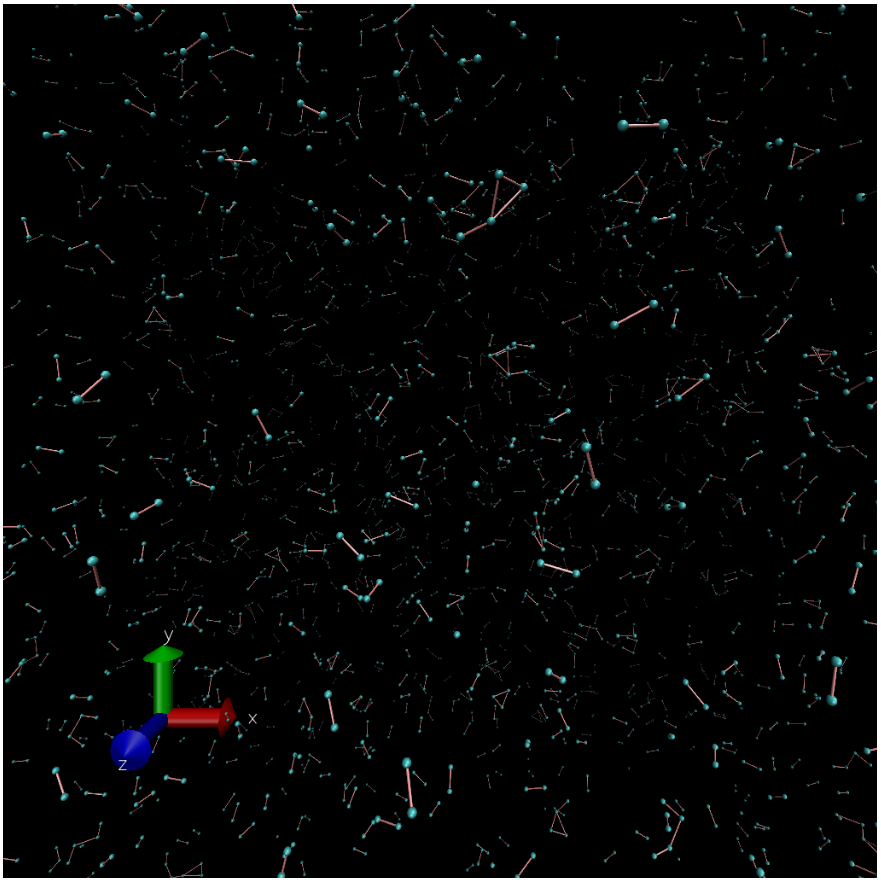

Figure S1: Snapshot of the dilute Te gas state (100 bar, 2760 K, 0.109 g/cm^3). Atoms are shown as spheres. A bond is shown for each pair of atoms closer than 4.2 Å.

We can also estimate the dimer oscillation frequency by a harmonic approximation around

the minimum of the Te_2 pair potential, as shown in Fig. S2. Note that because of the nature of the BOP, this pair potential is only valid when the pair is isolated from other atoms. The potential minimum is at $r = 2.737\,\text{Å}$ with a value of $-2.83\,\text{eV}$, or a disassociation energy of 65.3 kJ/mol, which is close to the 61 kJ/mol value mentioned above. The harmonic approximation is obtained by taking the second derivative of potential at its minimum, giving a value of $k = 9.554\,\text{eV/Å}^2$. Since each Te atom has a mass $m = 127.6\,\text{u}$, the oscillation frequency for the harmonic approximation is $\omega = \sqrt{2k/m} = 38.0\,\text{THz}$, or $\hbar\omega = 25.0\,\text{meV}$, close to the peak position in Fig. 1 in the main text and Fig. S5 below.

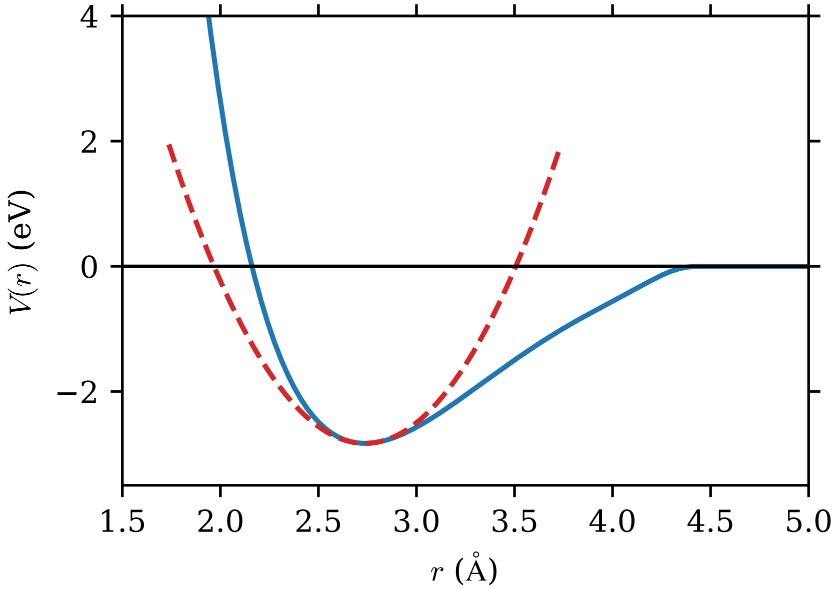

Figure S2: Solid line: potential energy of the Te_2 dimer as a function of the distance r between the atoms. Dashed line: harmonic approximation around the potential minimum.

Tellurium critical parameters

The liquid-gas critical point of the Te BOP has not been reported in the literature. Therefore, we obtain the Te critical parameters using direction MD simulations of the liquid-gas coexistence.[4] The simulation is done with 1080 atoms in a box with periodic boundary conditions. At each temperature, we first equilibrate using the NPT ensemble at $P = 1000\,\text{bar}$,

where the system is in the liquid state. The box size is approximately 70 Å × 25 Å × 25 Å after equilibration. Then, the box is expanded in the first dimension by 60 Å, and the simulation is run using the NVT ensemble for at least 2 ns with a time step of 2.5 fs. At each temperature, we fit the density profile along the first dimension with a hyperbolic tangent function for the gas-liquid interface[4] and obtain the bulk liquid and gas densities. At higher temperatures ($T \geq 1950\,\mathrm{K}$), the boundary is likely to move during the course of the simulation, so we average the density profile every 250 fs and fit each average with the hyperbolic tangent method.

The results are shown in Fig. S3a as red and blue crosses representing the densities of the gas phase and liquid phase, respectively. Their average is shown as grey crosses. These data are then fit assuming the law of rectilinear diameters and a scaling exponent for the density difference; i.e., let $\rho_\mathrm{G}(T)$ and $\rho_\mathrm{L}(T)$ represent the densities of the gas and liquid at temperature T, then

$$\frac{1}{2}\left[\rho_\mathrm{G}(T) + \rho_\mathrm{L}(T)\right] = \rho_c + k(T_c - T), \tag{1}$$

$$\rho_\mathrm{L}(T) - \rho_\mathrm{G}(T) = A\left(\frac{T_c - T}{T_c}\right)^{\beta}, \tag{2}$$

with ρ_c, T_c, k, A, and β as fit parameters. With fit results are shown in Fig. S3a with the black star indicating the position of the critical point: $\rho_c = (2.17 \pm 0.04)\,\mathrm{g/cm^3}$, $T_c = (2080 \pm 40)\,\mathrm{K}$. Incidentally, the scaling exponent $\beta = 0.32 \pm 0.03$ from the fit is consistent with the 3D Ising exponent of 0.33.[5] We then fit the pressure-temperature data shown in Fig. S3b to the Antoine equation[6] and get $P_c = (530 \pm 40)\,\mathrm{bar}$.

NMF components and dispersion relation

Figure S4 shows the G and L components obtained from the NMF fit. As expected for spectra in the free-particle limit, the G component shown on the left column has a single Gaussian-

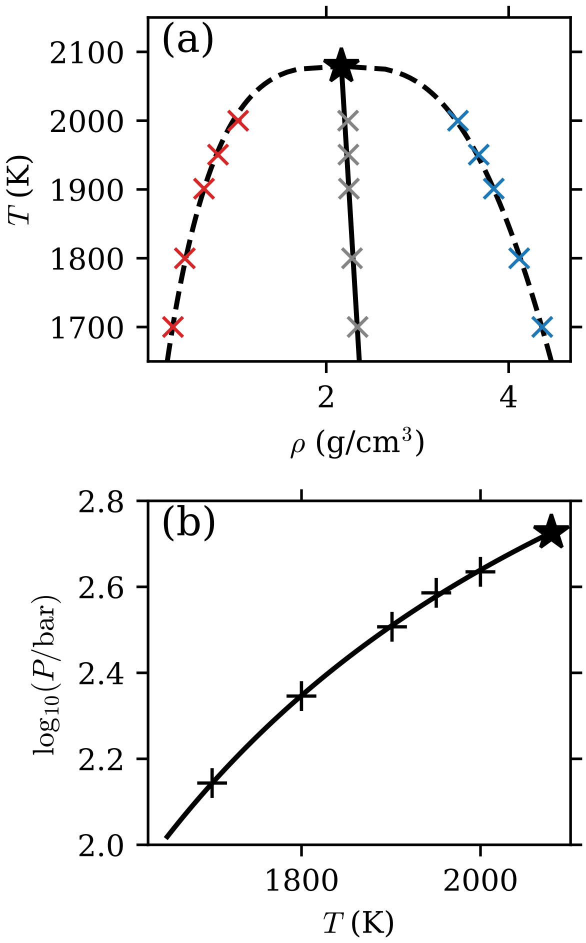

Figure S3: Simulation results for the liquid-gas coexistence curve of Te. (a) Densities of the gas (red crosses) and liquid (blue crosses) phase and their average (grey crosses); also shown are fit results for the law of rectilinear diameters (solid line), scaling of the density difference (dashed line), and critical point (black star). (b) Pressure-temperature data (black "+" symbols), best fit using the Antoine equation (solid line), and the critical point (black star).

like peak which broadens with increasing Q (except for Te with the extra dimer oscillation peak as discussed above). The peak position is expected to be $\sqrt{2}Qv_0 \propto Q$ according to Eq. (4) in the main text, and this is indeed the case as shown in Fig. S5. For Te, we can see in Figs. S4 and S5 that the dimer oscillation peak is dispersionless as expected.

The L component exhibits a more interesting behavior as shown on the right column of Fig. S4. For water, Si, and Te, the L component appears to contain two peaks in itself, and the one at lower frequency grows with increasing Q. This is reminiscent of previous MD simulation results on liquid water[7] where the two peaks were attributed to transverse and longitudinal modes. Here "transverse" and "longitudinal" refer to motions with a dominant transverse and longitudinal polarization, respectively. This is a plausible explanation for the shape of the L component shown here, and we note that the water, Si, and Te potentials all contain terms which give rise to local orientational order. In contrast, the LJ potential is merely pairwise and contains no orientational terms, and its L component does not show two distinct peaks either. However, in all cases, the low-frequency peak in the L component has significant overlap with the G component, making it difficult to distinguish between the two. Thus, the nature of the low-frequency peak cannot easily be determined with the approach used here and may be the subject of future investigations.

The high-frequency peak of the L component, on the other hand, can be directly observed in the $J_l(Q,\omega)$ spectra for all systems, and in the case of water it has been unanimously attributed to the longitudinal acoustic branch.[8] In Fig. S5 we plot the position of this high-frequency peak as blue dots. It can be seen that the peak frequencies is roughly proportional to Q up to the boundary of the pseudo-Brillouin zone,[8,9] which is typical of the behavior of liquids. The sound speeds corresponding to the linear relations are marked on Fig. S5. Note that in the case of water, because we have chosen a higher temperature range than in our previous work,[8] the sound speed extracted from the L component is somewhat lower, still fully consistent with the conclusions drawn in this study.

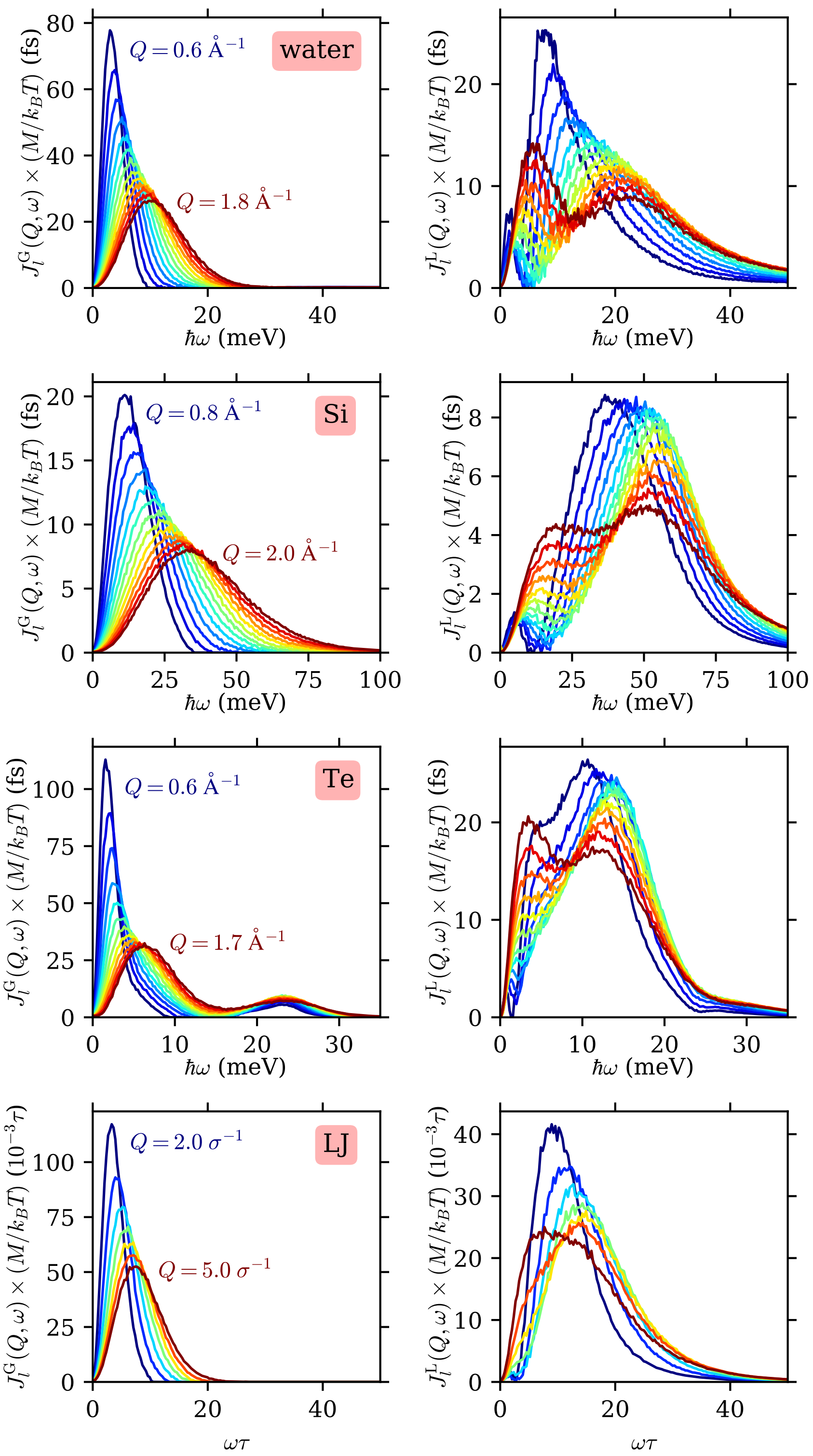

Figure S4: NMF components. Left (right) column shows the G (L) component for each system. From dark blue to dark red is increasing Q with uniform step size; the lowest and highest Q values are annotated in the plot.

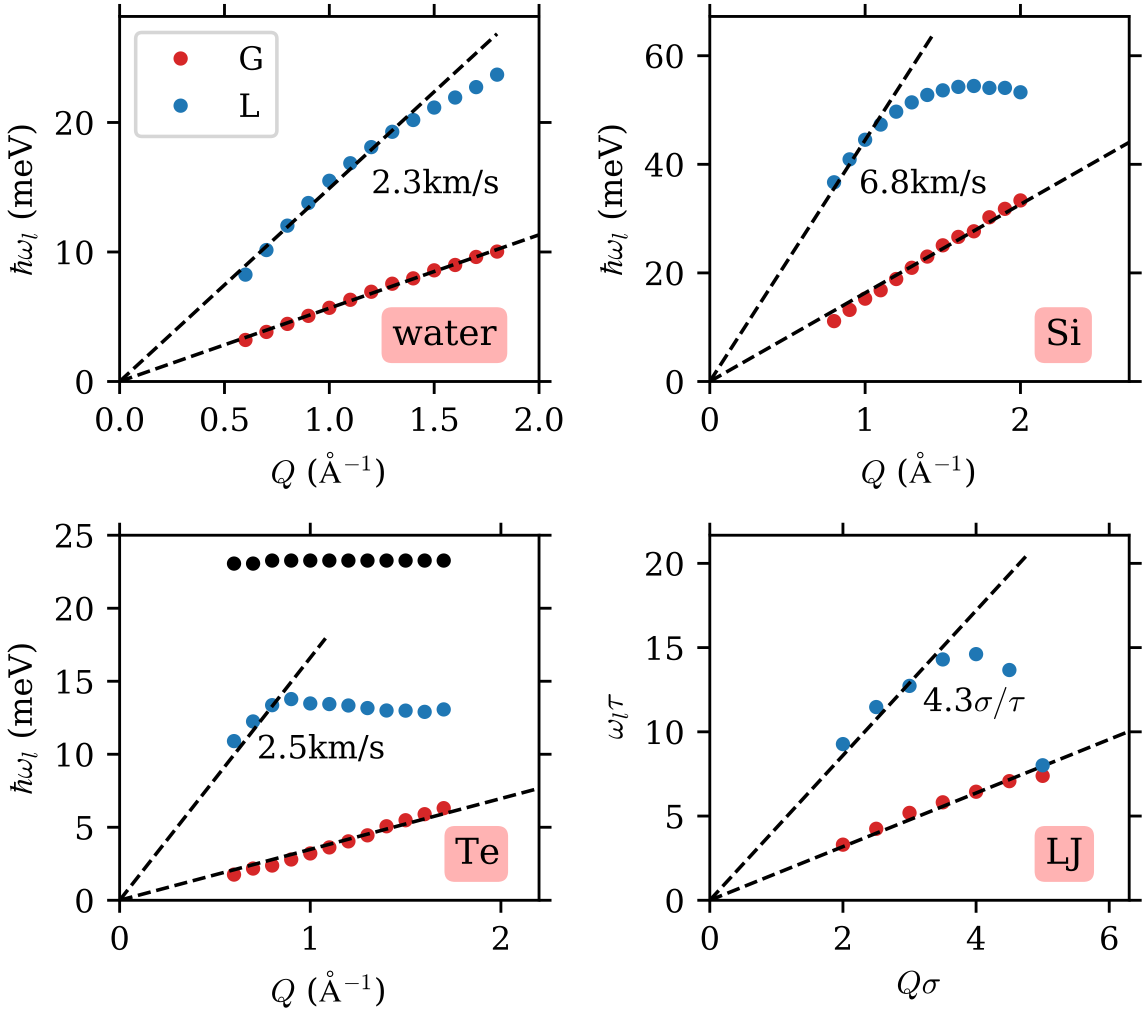

Figure S5: Peak frequency of the G (red) and L (blue) components as function of Q. For the L component of water, Si, and Te, only the higher-frequency peak position is plotted (see text). The dashed lines indicate linear relations. The sound speed corresponding to the dispersion relation of the L component is marked on the plot. For Te, the frequency of the dimer oscillation peak in the G component is shown as black dots.

Hydrogen bond definitions

A variety of criteria can be found in the literature to define hydrogen-bonding (H-bonding) in water, which result in different H-bond populations.[10–12] Here, as in our previous work,[8] we adopt the notations in Refs. 11 and 12. The variables used are defined in Fig. S6a. In the main text, we use a definition commonly found in the literature:[13,14] $R < 3.5\,\text{Å}$ and $\beta < 30°$, and we denote it here as the "R-β" criterion. Another definition, introduced in Ref. 11 and denoted here as "r-ψ", requires

$$(7.1 - 0.050\psi + 0.00021\psi^2)e^{-r/0.343} > 0.0085, \quad (3)$$

where r is in units of Ångströms and $\psi \in [0°, 90°]$ in degrees. This turns out to be among the most stringent definitions.[11,12] A rather relaxed one, denoted here as "r-R", requires $r < 2.5\,\text{Å}$ and $R < 3.5\,\text{Å}$.

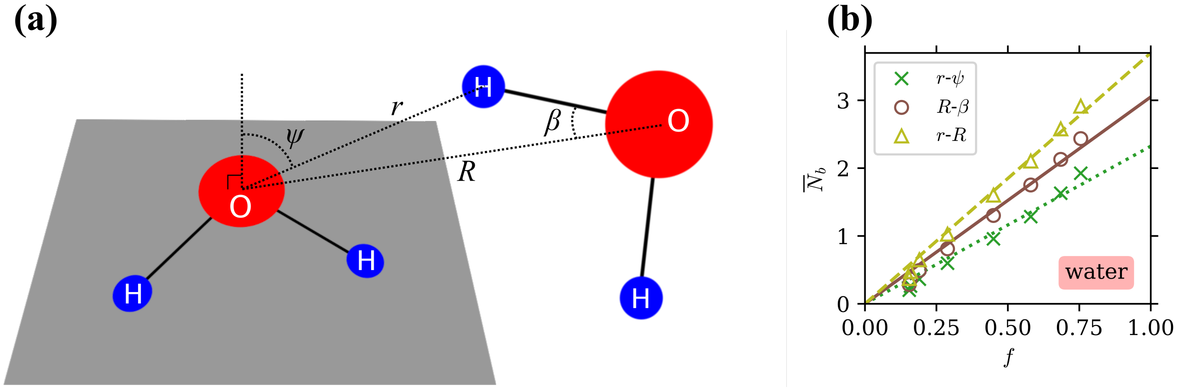

Figure S6: H-bond definitions. Panel (a) shows the variables defined. Panel (b) shows, for each of the three definitions detailed in the text, the average number of hydrogen bonds per molecule, $\overline{N}_b$, plotted again the parameter f.

In Fig. S6b we plot the average number of H-bonds per molecule, $\overline{N}_b$, against the parameter f using the definitions mentioned above. In all cases, there is good linearity between f and $\overline{N}_b$, and the data is consistent with an intercept at $\overline{N}_b = 0$.

RDF and cutoff for bonding definition

In Fig. S7 we show on the top row the radial distribution function (RDF), $g(r)$, for Si, Te, and LJ fluid, on the same isobar and temperature range as in the main text:

- Si — $P = 2850\,\mathrm{bar}$, T from 5200 K to 11 200 K in 600 K steps (omitting 10 600 K);
- Te — $P = 870\,\mathrm{bar}$, T from 1160 K to 2760 K in 160 K steps;
- LJ — $P = 0.13\varepsilon/\sigma^3$, T from $0.7\varepsilon/k_B$ to $1.3\varepsilon/k_B$ in $0.1\varepsilon/k_B$ steps.

The vertical lines show the locations of cutoff distances to define bonding. The solid line corresponds to the definition used in the main text, i.e., the first minimum in $g(r)$ at lower temperatures. In order to show that the conclusions do not depend sensitively on the exact cutoff distance, we also test changing it by ±10%, as indicated by the dashed and dotted lines.

On the bottom row, we plot the average number of bonds per atom, $\overline{N}_b$, against the parameter f using the different cutoff definitions. The linearity between $\overline{N}_b$ and f is good for all definitions (perhaps slightly better using the middle one, i.e., at the $g(r)$ minimum). The data are also consistent with an intercept at $\overline{N}_b = 0$ for Si and LJ and $\overline{N}_b = 1$ for Te, as discussed in the main text.

References

(1) Ward, D. K.; Zhou, X. W.; Wong, B. M.; Doty, F. P.; Zimmerman, J. A. Analytical bond-order potential for the cadmium telluride binary system. *Physical Review B* **2012**, *85*, 115206.

(2) Humphrey, W.; Dalke, A.; Schulten, K. VMD – Visual Molecular Dynamics. *Journal of Molecular Graphics* **1996**, *14*, 33–38.

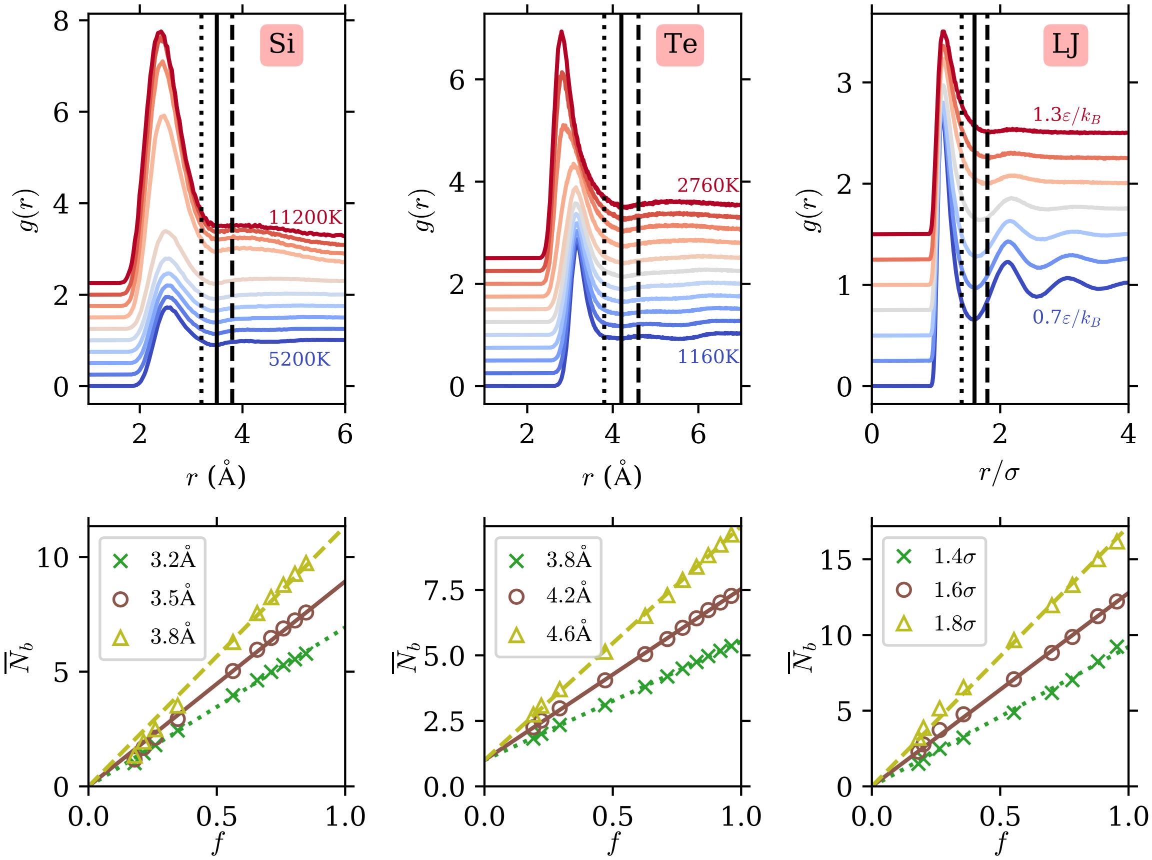

Figure S7: RDF and bonding definitions. Top row: RDF for Si, Te, and LJ fluid along the same isobars ($P = 2850\,\mathrm{bar}$, $870\,\mathrm{bar}$, $0.13\varepsilon/\sigma^3$, respectively) and temperature range (annotated on the plot) as in the main text. From dark blue to dark red are increasing temperatures, and an offset of 0.25 is added to $g(r)$ at each temperature step. Vertical lines show the cutoff definitions: solid line is the location of the first minimum in $g(r)$, and the dashed and dotted lines are approximately ±10% away. Bottom row: average number of bonds per atom, $\overline{N}_b$, plotted against the parameter f. Symbols represent the data using the cutoff shown in the top row; the exact values are shown in the legend. Lines show linear fits with a fixed intercept ($\overline{N}_b = 0$ for Si and LJ, 1 for Te).

(3) Budininkas, P.; Edwards, R. K.; Wahlbeck, P. G. Dissociation Energies of Group VIa Gaseous Homonuclear Diatomic Molecules. III. Tellurium. *The Journal of Chemical Physics* **1968**, *48*, 2870–2873.

(4) Alejandre, J.; Tildesley, D. J.; Chapela, G. A. Molecular dynamics simulation of the orthobaric densities and surface tension of water. *The Journal of Chemical Physics* **1995**, *102*, 4574–4583.

(5) Pelissetto, A.; Vicari, E. Critical phenomena and renormalization-group theory. *Physics Reports* **2002**, *368*, 549–727.

(6) Poling, B. E.; Prausnitz, J. M.; O'Connell, J. P. *Properties of Gases and Liquids, Fifth Edition*; McGraw-Hill Education: New York, 2001.

(7) Sampoli, M.; Ruocco, G.; Sette, F. Mixing of Longitudinal and Transverse Dynamics in Liquid Water. *Physical Review Letters* **1997**, *79*, 1678–1681.

(8) Sun, P.; Hastings, J. B.; Ishikawa, D.; Baron, A. Q.; Monaco, G. Two-Component Dynamics and the Liquidlike to Gaslike Crossover in Supercritical Water. *Physical Review Letters* **2020**, *125*, 256001.

(9) Giordano, V. M.; Monaco, G. Fingerprints of order and disorder on the high-frequency dynamics of liquids. *Proceedings of the National Academy of Sciences* **2010**, *107*, 21985.

(10) Matsumoto, M. Relevance of hydrogen bond definitions in liquid water. *The Journal of Chemical Physics* **2007**, *126*, 054503.

(11) Kumar, R.; Schmidt, J. R.; Skinner, J. L. Hydrogen bonding definitions and dynamics in liquid water. *The Journal of Chemical Physics* **2007**, *126*, 204107.

(12) Strong, S. E.; Shi, L.; Skinner, J. L. Percolation in supercritical water: Do the Widom and percolation lines coincide? *The Journal of Chemical Physics* **2018**, *149*, 084504.

(13) Luzar, A.; Chandler, D. Hydrogen-bond kinetics in liquid water. *Nature* **1996**, *379*, 55–57.

(14) Luzar, A.; Chandler, D. Effect of Environment on Hydrogen Bond Dynamics in Liquid Water. *Physical Review Letters* **1996**, *76*, 928–931.
